# Aberration in myeloid-derived pro-angiogenic cells in type-2 diabetes mellitus; implication for diabetic retinopathy?

**DOI:** 10.3389/fopht.2023.1119050

**Published:** 2023-03-23

**Authors:** Mahnaz Shariatzadeh, Trishika R.R. Binda, Conny van Holten-Neelen, Josianne C. ten Berge, Jose P. Martinez Ciriano, King T. Wong, Willem A. Dik, Pieter J.M. Leenen

**Affiliations:** ^1^ Department of Immunology, Erasmus University Medical Center, Rotterdam, Netherlands; ^2^ Department of Immunology, Laboratory Medical Immunology, Erasmus University Medical Center, Rotterdam, Netherlands; ^3^ Department of Ophthalmology, Erasmus University Medical Center, Rotterdam, Netherlands; ^4^ Rotterdam Eye Hospital, Rotterdam, Netherlands

**Keywords:** diabetic retinopathy, myeloid-derived pro-angiogenic cells, retinal endothelial cells, *in vitro* angiogenesis, microvascular dysfunction

## Abstract

**Purpose:**

Diabetic retinopathy (DR) is a major microvascular complication of type 2 diabetes mellitus (T2DM). Myelomonocytic proangiogenic cells (PAC) have been implicated in DR pathogenesis, but their functional and developmental abnormalities are unclear. In this study we assessed PAC characteristics from healthy controls, T2DM patients with DR (DR) and without (NoDR) in order to determine the consequence of the diabetic condition on PAC phenotype and function, and whether these differ between DR and NoDR patients.

**Methods:**

PAC were generated by culturing PBMC on fibronectin coating and then immunophenotyped using flow cytometry. Furthermore, cells were sorted based on CD14, CD105, and CD133 expression and added to an *in vitro* 3-D endothelial tubule formation assay, containing GFP-expressing human retinal endothelial cells (REC), pericytes, and pro-angiogenic growth factors. Tubule formation was quantified by fluorescence microscopy and image analysis. Moreover, sorted populations were analyzed for angiogenic mediator production using a multiplex assay.

**Results:**

The expression of CD16, CD105 and CD31, but not CD133, was lower in PAC from T2DM patients with or without DR. Myeloid and non-myeloid T2DM-derived sorted populations increased REC angiogenesis *in vitro* as compared to control cultures. They also showed increased S100A8 secretion, decreased VEGF-A secretion, and similar levels of IL-8, HGF, and IL-3 as compared to healthy control (HC)-derived cell populations.

**Conclusion:**

T2DM PAC are phenotypically and functionally altered compared to PAC from HC. Differences between DR and NoDR PAC are limited. We propose that impaired T2DM PAC provide inadequate vascular support and promote compensatory, albeit pathological, retinal neovascularization.

## Introduction

Angiogenesis, i.e. the formation of new capillaries from pre-existing blood vessels, plays an ambiguous role in the pathogenesis of vascular dysfunction in diabetes mellitus (DM) (1). Diabetic retinopathy (DR) is a serious ocular complication of DM characterized by excessive angiogenesis in the retina, especially in the late stage of the disease. It is one of the main manifestations of microvascular dysfunction in type 2 diabetes mellitus (T2DM) and leads to eye hemorrhages and vision loss. Although DR is thought to be initiated by local tissue inflammation and hypoxia (2), systemic factors influencing immune cell function and their interaction with endothelial cells (EC) seem to play a significant role as well.

Myeloid cells, in particular monocytes and macrophages, closely interrelate with EC and contribute to vascular homeostasis in both healthy and diseased situations (3). Myeloid-derived pro-angiogenic cells (PAC), formerly termed “endothelial progenitor cells” or circulating angiogenic cells, are believed to have a supportive effect on vascular homeostasis (4). The monocytic origin of PAC is confirmed by several studies (5–7), although approaches for generating these cells *in vitro* from peripheral blood mononuclear cells (PBMC) differ between studies, as do the functional and phenotypic definitions (4, 8–10). Nonetheless, most investigators in the field classify PAC as a subset of monocyte-derived cells with CD14^+^CD16^+^ phenotype, which co-express hematopoietic and endothelial markers and possess M2-macrophage-like features (11). A deficit in the formation of these cells has been reported previously in diabetes, in association with diabetic vascular dysfunctions (12, 13). This, on the one hand, is in line with the macrovascular dysfunction in diabetes, but on the other hand is seemingly contradictory to the excessive, dysregulated angiogenesis in DR. Therefore, it is vital to shed more light on the biology of PAC in terms of their phenotype and angiogenic properties in DR. This might give a better understanding of the aberrant retinal vessel formation in DR and eventually provide targets for therapy.

In this study, we focus on the phenotype and pro-angiogenic capacity of PAC in patients with T2DM with or without DR. Specifically, we approach the question whether PAC from T2DM patients with or without DR are phenotypically and functionally different from healthy PAC, and whether these alterations correlate to the microvascular dysfunctions in DR. To this end, we determine the immunophenotype of PAC using flow cytometry-based detection of monocyte marker CD14, stem/progenitor cell marker CD133, and angiogenic markers CD105 and CD31, as identifying parameters for genuine PAC (11). Alongside, we assess the pro-angiogenic function of PAC in a 3-D tubule formation assay of retinal endothelial cells (REC), and evaluate PAC for the production of a selected panel of angiogenic mediators, with the final goal of increasing our understanding of the biology of PAC in DR.

## Materials and methods

### Study design and patients

Patients with T2DM referred to the Rotterdam Eye Hospital or the Department of Ophthalmology, Erasmus MC, were included and, based on their clinical diagnosis, assigned to one of the following groups: T2DM without DR (NoDR), with non-proliferative DR (NPDR) or with proliferative DR (PDR). This diagnosis was established according to the international disease severity scale based on the early treatment diabetic retinopathy study (ETDRS) classification (14). Healthy controls (HC) were recruited from blood bank donors (Sanquin, Amsterdam, the Netherlands) and hospital staff. HC did not have a history of systemic inflammation or inflammatory eye disease and were not under any specific diet or medication. In accordance with ADA- and WHO- guidelines, diabetes was defined as a fasting plasma glucose ≥ 7.0 mmol/L and/or a non-fasting plasma glucose level ≥ 11.1 mmol/l and/or treatment with oral glucose-lowering medication or insulin or the diagnosis of T2DM registered by a medical specialist. Patients with type 1 diabetes mellitus or other types of DM were excluded from the study. Written informed consent was obtained from all participants.

T2DM patients without DR (NoDR) did not have retinal alterations seen with ophthalmoscopy. T2DM patients with NPDR displayed more than one of the following retinal alterations: microaneurysms, blood, hard exudates, cotton wool spots, venous looping or beading, and IRMAs (intraretinal microvascular abnormalities). T2DM patients with PDR exhibited vitreous or pre-retinal hemorrhage with neovascularization of the disc or elsewhere. The study was approved by the local medical ethics committee of Erasmus MC (MEC-2018-148 and MEC-2016-202) and conducted in accordance with the ethical principles of the Declaration of Helsinki. Demographic data of the study cohort are summarized in **Table 1**.

**Table 1:** Demographic data from the participants included in the study.

Demographics	T2DM + NoDR (n = 13)	T2DM + DR (n = 48)	Healthy control (n = 17)
**Male, n (%)**	10 (77%)	28 (58%)	Not available
**Age range in years, mean ± SD**	59 – 84, 75 ± 9.5	48 – 91, 69 ± 10	Not available
**BMI (mean ± SD)**	29.4 ± 4.3	29.7 ± 5.9	Not available
**Disease duration in years,** **mean ± SD**	8 – 22,16 ± 6	9 – 40,18 ± 8	Not applicable
**Insulin injection**	2 (15%)	21 (45%)	Not applicable

### PBMC isolation

Human peripheral blood from participants was collected in heparin tubes and peripheral blood mononuclear cells (PBMC) were isolated through standard Ficoll-Paque gradient centrifugation (15). PBMC were transferred into new tubes, washed with phosphate-buffered saline (PBS), and centrifuged (10 min at 760 × g). Cell pellets were resuspended in RPMI freezing medium containing 40% fetal bovine serum (FBS, Gibco) + 10% dimethyl sulfoxide (DMSO, Sigma-Aldrich, St Louis, MO, USA) and stored in liquid nitrogen until further use.

### *In vitro* PAC formation

PBMC isolated from patients and healthy donors were thawed and resuspended in M199, Earle’s Salts medium (Gibco™, cat # 11150059) supplemented with 2% FBS and then plated on fibronectin-coated 12-well culture plates (2 × 10^6^ cells/ml per well) or 48-well culture plates (0.4 × 10^6^ cells/200 µl per well) for cell sorting and flow cytometric phenotype analysis, respectively. After 4 days of culture, cell morphology was examined at 20× magnification using an Axiovert 100 light microscope (Zeiss, Oberkochen, Germany). Next, the culture supernatant was discarded and the adhered cells were harvested using 30 minutes of incubation with cold PBS + 1 mM EDTA. Harvested cells were washed with staining buffer (MACSima™ running buffer, Miltenyi Biotec, Bergisch Gladbach, Germany) and exposed to optimally titrated anti-human monoclonal fluorescent antibodies at 4°C in a dark environment, according to the manufacturer’s instructions. The following antibodies were used: CD14-PE-Cy7 (61D3, eBioscience™), CD16 (FcγRIII)-APC-Cy7 (3G8 (RUO), BD Pharmingen™), CD105 (endoglin)-APC (166707, R&D systems), CD133-PE (170411, R&D systems), CD163-PerCP (GHI/61, eBioscience™), CD31 (PECAM-1)-PerCP (WM59, BioLegend), anti-HLA-DR (MHC class II)-FITC (G46-6, BD Pharmingen™) and CD206-FITC (15-2, MRC1; BioLegend). For cell sorting, the cells were stained with fluorescent CD14, CD105, and CD133 antibodies. After 20 minutes of incubation, the cells were washed and resuspended in 200 µl staining buffer.

### PAC detection

Surface marker expression was measured using a FACScanto™II cell analyzer (BD Biosciences, Piscataway, NJ, USA). Viable cells were gated and data were analyzed using FlowJo software (Tree Star, Ashland, OR, USA). At least 10,000 events were acquired per sample (gating strategy is available in **Figure S1**). Cell sorting was performed using a FACSAria-III machine. Based on CD14, CD105, and CD133 expression we sorted the following cell populations (1): all cells (total live singlets as total PBMC-derived population), (2) non-myeloid CD14^-^ (CD14^-^CD105^-^CD133^-^), (3) myeloid CD14^+^DN (CD14^+^CD105^-^CD133^-^) and (4) myeloid CD14^+^DP (PAC, CD14^+^CD105^+^CD133^+^) both directly on top of the wells of the tubule assay plate (500 cells/well) and in separate FACS tubes (10^5^ cells/tube) for further overnight culture (**Figure S2**).

### 3-D *in vitro* tubule formation assay

To explore the effect of PAC on REC vessel formation we made use of a three-dimensional retinal tubule formation assay as described previously (16). Briefly, GFP-expressing human REC (cat # ACBRI 181, Cell Systems, Troisdorf, Germany) were co-cultured with human brain vascular pericytes (cat # SCC1200-KIT, Sciencell research, San Diego, USA) in a collagen matrix (bovine collagen type-1, Gibco™, cat # A1064401) in the presence of three pro-angiogenic growth factors (interleukin (IL)-3, stem cell factor (SCF), and stromal cell-derived factor-1α (SDF-1α); R&D Systems, Abingdon, UK; each at 25 or 200 ng/ml as indicated in the text. After 24 hours, successful sprouting of EC was typically observed and then sorted PAC subpopulations were added on top of the wells in four replicates. The tubule formation of the REC was imaged 4 days after the initiation of the culture, hence 3 days after adding the sorted PAC populations, using 20x magnification of an inverted fluorescence microscope (Olympus SC30, Shinjuku, Japan), and the total surface area was quantified by FIJI software (version 1.51n) (16).

### Pro-angiogenic factor measurement

To assess the production capacity of pro-angiogenic factors, sorted subpopulations derived from the PAC cultures were kept overnight in 2% BSA-containing RPMI medium (10^5^ cells/100 µl) in a round bottom 96-well culture plate. Production of the pro-angiogenic factors FABP4, HGF, IL-3, IL-8, S100A8, SCF, SDF-1α and, VEGF-A was measured using a Luminex multiplex bead immunoassay (R&D systems, Abdingdon, UK), according to the manufacturer’s instruction. The data acquisition was performed on a BioPlex MAGPIX machine and data were analyzed applying Bio-Plex Manager MP software (Bio-Rad, Hercules, California, USA).

### Statistics

All statistical analyses were performed using GraphPad Prism (version 5.04). To identify significant differences between different experimental conditions, student T-test, non-parametric Mann Whitney U T-test, or one-way ANOVA followed by a *post-hoc* Bonferroni or Tukey multiple comparison test were used, depending on normality of the obtained data. A *P* value < 0.05 was considered to indicate a statistically significant difference. All data are presented as means ± standard error of the mean (SEM). A principal component analysis (PCA) biplot was created using the prcomp() function in R Studio version 4.2.1, and the ggbiplot2 package was applied to visualize the results.

## Results

### Monocytes from patients with T2DM show altered PAC formation compared to healthy controls

**Figure 1:**
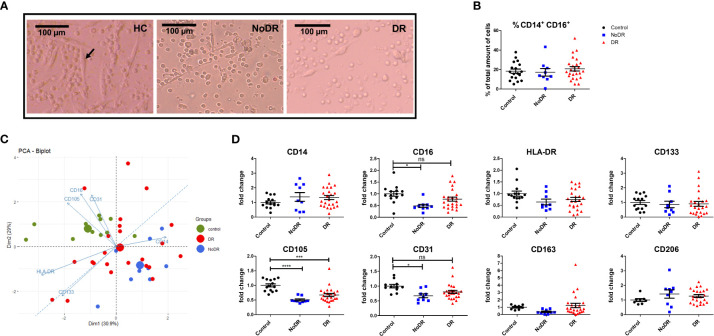
PAC from patients with DR display an altered phenotype compared to controls. PBMC were isolated from healthy controls (HC, n = 14), patients with T2DM without retinopathy (NoDR, n = 9), and patients with T2DM with retinopathy (DR, n = 24). Monocytes were stimulated for 4 days to differentiate into PAC. **(A)** Bright field microscopy of spindle-shaped PAC (arrow) from HC, NoDR and DR patient PBMC, 150× magnification. **(B)** Percentage CD14^+^CD16^+^ cells, indicating frequency of monocyte-derived cells after culture. **(C)** Principal component analysis (PCA) biplot based on median fluorescent intensities (MFI) of CD14, CD16, CD105, CD31, CD133, and HLA-DR as measured on PAC from HC, NoDR and DR patients. Small dots represent each donor, large dots show the average. **(D)** Individual marker profiles of PAC. Values were corrected for auto-fluorescence by subtracting the backbone control, and normalized to the average of expression by HC PAC. Error bars represent means ± standard error of the mean (SEM). Significance was calculated using one-way ANOVA and Bonferroni *post-hoc* test correction for multiple comparisons. ns, non-significant, *p < 0.05, ***p < 0.001, ****p < 0.0001.

We generated monocyte-derived PAC with the characteristic spindle-shape morphology from 33 T2DM patients and 14 controls (black arrow in **Figure 1A**). Although the frequencies of monocyte-derived cells were comparable between the groups following culture (**Figure 1B**), formation of spindle-shaped cells was reduced apparently in both T2DM patients with and without DR, in comparison with healthy controls.

Altered PAC development in T2DM was confirmed phenotypically by Principle Component Analysis (PCA) based on expression of six surface markers (CD14, CD16, CD105, CD31, CD133 and HLA-DR). This clearly separated T2DM PAC from HC, and with more distance of the NoDR than the DR group from HC (**Figure 1C**). This could be primarily attributed to decreased expression of CD16 (*P* = 0.01) as well as of endothelial markers CD105 (*P* < 0.0001) and CD31 (*P* = 0.01) that was significantly lower among CD14+ monocyte-derived cells in patients with T2DM compared to controls (**Figure 1D**). There was, however, no clear distinction between PAC from NoDR, NPDR and PDR patients (**Figure S3**). Expression of PAC marker CD133, M2-macrophage markers CD163 and CD206, as well as HLA-DR did not differ between PAC from T2DM patients and HC PAC. Accordingly, we conclude from these data that PAC generated from patients with T2DM have an altered phenotype compared to those generated from non-diabetic controls.

### Monocyte-derived PAC from T2DM patients and HC differentially affect *in vitro* angiogenesis

**Figure 2:**
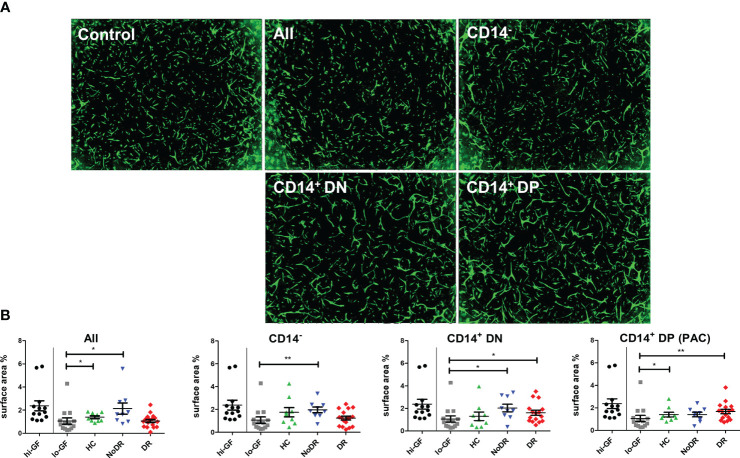
PAC culture-derived cells from patients with T2DM with and without DR enhance *in vitro* angiogenesis. PBMC isolated from HC (n = 9), NoDR (n = 9), and DR patients (n = 17) were cultured to differentiate into PAC. The total cell culture population (All), or flow-sorted subsets (CD14^–^CD105^–^CD133^–^ (CD14^–^), CD14^+^CD105^–^CD133^–^ (CD14^+^DN), or CD14^+^CD105^+^CD133^+^ (CD14^+^DP, PAC)) (500 cells) were introduced into a gelatin-based 3-D *in vitro* tubule formation assay of retinal endothelial cells (REC) and pericytes supplemented with low concentrations of pro-angiogenic growth factors (lo-GF). After 4 days, REC tubule formation was visualized by fluorescence microscopy. **(A)** Representative images from co-cultures stimulated with different sorted cell subsets from a DR patient. ‘Control’ indicates the angiogenesis observed in a lo-GF culture. **(B)** Quantification of angiogenesis represented as total REC surface area percentage. Each experiment included culture quality controls with high growth factor concentration **(hi-GF)** and lo-GF, and tested different cell subsets from HC and patients in lo-GF condition. Dots show data from individual cultures (average of at least 4 technical replicates) in separate experiments normalized to the average of lo-GF control. Error bars represent means ± standard error of the mean (SEM). Significance was calculated compared to the lo-GF control using Mann Whitney U T-test. *p < 0.05, **p < 0.01.

The pro-angiogenic capacity of myeloid-derived PAC from HC and T2DM patients with or without DR was examined in an *in vitro* 3-D tubule formation model specifically adapted to assess REC angiogenesis (16). To that end, three subpopulations of cells were sorted from T2DM patient- and HC-PAC cultures (**Figure S2**) based on their differential expression of CD14, CD105, and CD133. Also the total PBMC-derived population, comprising both lymphoid and monocyte-related cells, was sorted and assessed. These flow-sorted PBMC culture-derived populations (i.e. CD14^–^CD105^–^CD133^–^ (CD14^–^), CD14^+^CD105^–^CD133^–^ (CD14^+^DN) and CD14^+^CD105^+^CD133^+^ (CD14^+^DP – PAC)) were added to sprouting REC – pericyte co-cultures, and stimulated with low concentrations of angiogenic growth factors (lo-GF) (**Figure 2**). As a control for optimal angiogenesis, REC cultures were stimulated with high concentrations of growth factors (hi-GF) without exogenous cells. This showed an approximate 2.5 –fold increase in tubule formation. From the three sorted populations harvested from PBMC cultures, all T2DM-derived myeloid populations (i.e. CD14^+^CD105^–^CD133^–^ (CD14^+^DN) and CD14^+^CD105^+^CD133^+^ (CD14^+^DP PAC)), increased REC *in vitro* tubule formation as compared to control lo-GF cultures without cells added, although addition of CD14^+^DP cells from NoDR patients did not reach statistical significance (**Figure 2**). Considering myeloid-derived cells from HC, we only found cells with a genuine PAC phenotype (CD14^+^DP) to stimulate tubule formation significantly. Further, we observed no significant difference between NoDR and DR patients in the pro-angiogenic function of myeloid-derived cells. Together, these data suggest that myeloid-derived populations from T2DM patients’ PBMC cultures promote angiogenesis by REC *in vitro* to a greater extent than myeloid-derived cells from healthy controls, although the relatively high degree of variation and small sample size did not reveal significance in a direct comparison between the groups.

### Disturbed angiogenic mediator production by myeloid-derived cells from patients with T2DM, in particular with DR

**Figure 3:**
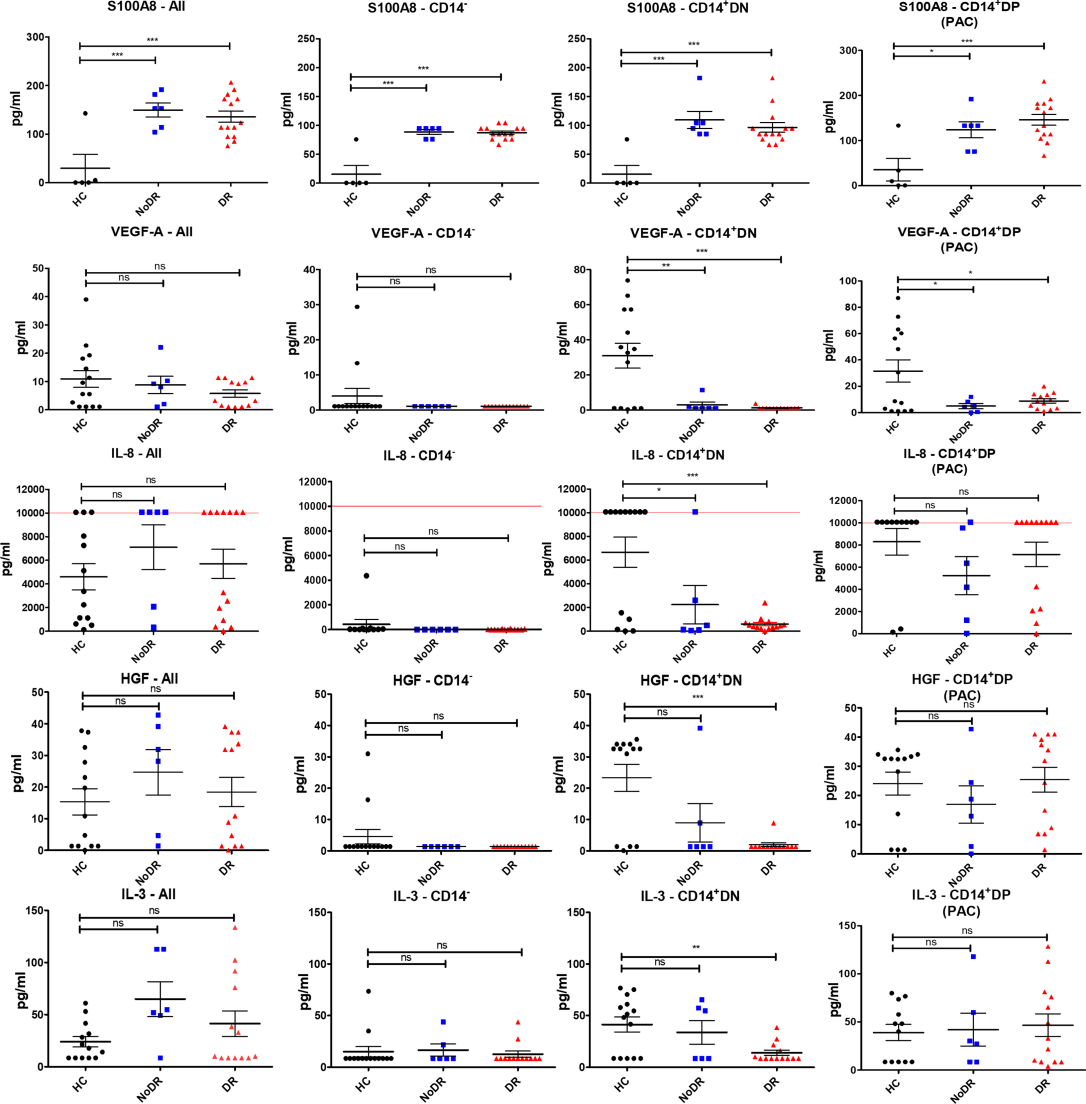
Myeloid-derived cells from patients with DR display the greatest imbalance in pro-angiogenic cytokine production compared to HC. PBMC isolated from HC (n = 14), NoDR (n = 6), and DR patients (n = 14) were cultured to differentiate into PAC. Four populations were isolated by flow cytometry: total live cells (All), lymphoid CD14^–^CD105^–^CD133^–^ (CD14^–^), and myeloid CD14^+^ CD105^–^CD133^–^ (CD14^+^ DN), and CD14^+^ CD105 ^+^ CD133 ^+^ (CD14^+^ DP, PAC) cells. From each population 10E5 cells were cultured for 24 hours and conditioned media were tested for cytokine production using a multiplex assay. The experiments were performed in two technical replicates for each patient. Values above/below the limit of detection are assigned at the highest/lowest limit of detection of the kit. Error bars represent means ± standard error of the mean (SEM). Significance was calculated compared to the control using one-way ANOVA followed by *post-hoc* Tukey multiple comparison test. ns, non-significant, *p < 0.05, **p < 0.01, ***p < 0.001.

Next, we assessed whether the pro-angiogenic properties of cultured PBMC populations are reflected in differential proangiogenic mediator production (**Figure 3**). After another 24h culture of flow-sorted cells, we detected measurable concentrations of S100A8, VEGF-A, IL-8, HGF and IL-3 in culture supernatant (FABP4, SCF, and SDF-1α were not detected). Myeloid-derived cells, i.e. CD14^+^DN and CD14^+^DP were the predominant producers of these cytokines. In particular, we detected a significant increase in the levels of S100A8 in cultures of T2DM patient cells, regardless of disease group and/or cell population (i.e., in both CD14^+^ and CD14^–^ populations (*P* < 0.001)) (**Figure 3**). We further identified that both CD14^+^ myeloid populations isolated from T2DM patients produced less VEGF-A (*P* < 0.01 for CD14^+^DN and *P* < 0.05 for CD14^+^DP) compared to HC. Similarly, CD14^+^DN cells from patients with diabetes produced significantly less IL-8 (*P* < 0.05 for comparison to NoDR; *P <* 0.001 for DR patients), while HGF and IL-3 secretion was only significantly less in the DR group (*P* < 0.001, and *P* < 0.01, respectively). It is remarkable that production of IL-8, HGF and IL-3 by CD14^+^DP PAC appeared rather well retained in T2DM patients, while production by CD14^+^DN myeloid cells is strongly affected, in particular in DR patients. Notably, in cultures of CD14^+^DP and CD14^+^DN cells from HC as well as T2DM patients, cytokine secretion is detected in most but not in all samples. No major differences were observed between NoDR and DR samples in cytokine production by myeloid cells. Together, these data show a shift in angiogenic factor production by myeloid-derived cells in T2DM patients with a decrease in VEGF-A, and a strong increase in the inflammatory mediator S100A8.

## Discussion

DR is characterized by retinal vessel abnormalities, indicative of disturbed endothelial homeostasis (17). The proper functionality of myeloid-derived PAC is very important for vascular integrity (13). Here we demonstrate that PAC generated from peripheral blood monocytes from patients with T2DM with or without DR are phenotypically altered compared to PAC from HC. Moreover, distinct populations of cells isolated from PAC cultures from patients with T2DM stimulated *in vitro* retinal vessel formation more strongly than those obtained from HC. This altered functionality was reflected in a modified angiogenic factor secretion profile, in particular, characterized by strongly elevated secretion of the inflammatory mediator S100A8. In general, only minor differences existed between PAC culture-derived cells from T2DM patients with or without DR.

Our data show that PAC differentiated from PBMC from patients with T2DM with or without DR are immunophenotypically different from PAC obtained from HC. More specifically, this is evidenced by reduced expression of CD16, CD105, and CD31. We interpret this as an indication of deviated PAC differentiation under diabetic conditions, which might contribute to vessel pathology in T2DM. Our findings of aberrant PAC differentiation are in line with previous studies that showed diminished proliferative capacity by T2DM PAC as well as a reduced frequency of circulating pro-angiogenic monocytes expressing CD14 or CD163 (18, 19). Together with a shift from M2 to M1 phenotype in patients with T2DM, these studies suggest an increased inflammatory profile of monocyte-derived PAC in diabetes (18–20). Van Ark et al. reported a reduced number of PAC in patients with diabetes irrespective of the presence or absence of macrovascular complications (21), but Tecilazich et al, found no change in the PAC count in uncomplicated diabetes (22), which is similar to the finding in our study. The reason why PAC differentiation is poor in diabetes is not known yet, however, its reduced proliferation in diabetic conditions is correlated with HbA_1c_ levels and might be attributed to the loss of tight glucose control (18).

Altered PAC differentiation and functionality in T2DM is further supported by our data demonstrating increased *in vitro* tubule formation of REC supplemented with PAC generated from patients with DR. In addition, all CD14^+^ cells derived from patients with T2DM with or without DR, and also CD14^-^ cells from patients with T2DM without DR stimulated REC angiogenesis. This suggests that, in diabetic conditions, not only PAC but also other immune cells such as lymphocytes are primed in a pro-angiogenic manner.

To further evaluate the angiogenic capacity of PAC, we measured the secretion of a number of angiogenic factors in PAC generated from patients and HC and found that this is altered in T2DM PAC. Most remarkably, we found the pro-inflammatory and pro-angiogenic factor S100A8 to be significantly higher expressed by all PAC culture-derived cell populations from T2DM patients with and without DR. S100A8 is a myeloid-related damage-associated molecular pattern and its increased production illustrates the imbalanced inflammatory and angiogenic response in T2DM. Increased expression of S100A8 has been previously reported in macrophages in inflamed tissues, and also in patients with T1DM and T2DM, particularly in those with retinopathy and nephropathy (23–26). Interestingly, S100A8 expression recently has been linked to DR progression (25). IL-1β can induce the expression of S100A8, and IL-6 and TNF-alpha correlate positively with S100A8 expression and diabetic vascular dysfunction (27, 28). It has been shown that in hypoxic conditions, HIF-1α-induced expression of S100A8 in monocytes stimulates angiogenesis (29). Although S100A8 is well known for its pro-angiogenic capacity (30, 31), a previous study reported that S100A8, at concentrations secreted by CD34^-^/CD31^+^ PAC inhibits HUVEC tubule formation *in vitro* through TLR4 activation (32). This contradiction might be due to the hormetic effects of the protein at low and high concentrations as suggested by the same study. We also found that T2DM PAC secrete significantly less VEGF-A, but similar amounts of the pro-angiogenic cytokines HGF, IL-3, and IL-8 compared to PAC from HC. All of these cytokines contribute to capillary sprouting and vascular density (33). Release of pro-angiogenic cytokines such as G-CSF, HGF, IL-8, PlGF, TGF-β, and VEGF as well as a few anti-angiogenic factors by PAC of myeloid origin has been previously reported (34–36).

Increased pro-angiogenic properties of PAC have been extensively studied before (4, 37, 38), nonetheless, the angiogenic function of PAC in diabetes is poorly understood. Several studies indicated impaired functionality of PAC in diabetes including proliferation, adhesion, and incorporation into vascular structures (18, 39). The increased pro-angiogenic capacity of our T2DM PAC might be related to the heterogeneity in EC types as we, for the first time, applied REC, which are the most affected cells in retinopathy, and may respond differently to ischemic events compared to other EC types.

One limitation of our study is that the HC were not age-matched to the patient groups. However, the PAC functional parameters measured in our study did not seem to be age-related among our patients. Another limitation of our study is measuring only a biased, small number of angiogenic mediators. Nonetheless, using REC in the functional assays, and detailed functional testing of cells with PAC activity are the strengths of the present study.

In conclusion, considering the important role of PAC in both vessel homeostasis and wound repair, it is tempting to speculate that healthy PAC support healthy neovascularization by balancing pro- and anti-angiogenic mediators, while this balance is disrupted in PAC under hyperglycemic and hypoxic conditions. This hypothesis correlates to the *in vivo* situation, as many patients with T2DM have defective blood vessel formation after ischemic events, but develop retinal neovascularization (18). Moreover, our data support the view that PAC are disturbed in T2DM and as such provide inadequate vascular support and promote compensatory, albeit pathological, retinal neovascularization. We did not find major differences between PAC derived from T2DM patients with or without DR, suggesting that microvascular conditions in NoDR are at risk, but compensated, while in DR these are decompensated and lead to pathology. Our results do not indicate specific switches in monocytes/macrophages or PAC functionality between these conditions. The question of how phenotypically deviant T2DM PAC with low expression of endothelial markers can have increased pro-angiogenic activity is intriguing. A plausible explanation would be that other factors than the few that are measured here might have a dominant effect. Further studies will be necessary to focus on a comprehensive evaluation of pro- and anti-angiogenic cytokines released by myeloid-related PAC generated from patients with T2DM and in relation to DR.

## Data availability statement

The original contributions presented in the study are included in the article/**Supplementary Material**. Further inquiries can be directed to the corresponding author.

## Ethics statement

The study was approved by the local medical ethics committee of Erasmus MC (MEC-2018-148 and MEC-2016-202). The patients/participants provided their written informed consent to participate in this study.

## Author contributions

MS is the first and corresponding author of this paper, performed most parts of the project and wrote the paper. TB contributed in the experimental part of the project, and R analysis. CH-N contributed in the experimental part of the project. JB provided patient samples and reviewed the manuscript. JM provided patient samples and reviewed the manuscript. KW provided patient samples and reviewed the manuscript. WD supervised the project and revised the paper. PL supervised the project and revised the paper. All authors contributed to the article and approved the submitted version.

## Supplementary material

Supplementary Figure 1 Gating strategy for flow cytometric analysis of myeloid-derived pro-angiogenic cells (PAC). **(A)** Forward scatter (FSC) x side scatter (SSC) gating to obtain mononuclear cells based on size and granularity and exclude debris. **(B)** cell aggregate exclusion. **(C)** gate for CD14^+^ monocyte-derived cells. **(D-H)** The representative marker expressions are plotted against the CD14 expression. Identical gates were used for auto-fluorescence compensation.

Supplementary Figure 2 Gating strategy for sorting cell subsets from PAC cultures. **(A)** Forward scatter (FSC) x side scatter (SSC) gating to obtain mononuclear cells based on size and granularity (P1). **(B)** Cell aggregate exclusion (P2). **(C)** Outline of gating strategy for myeloid and lymphoid cells. (D) gates for CD14^+^ cell subsets based on CD105 and CD133. CD14^+^DN refers to CD14^+^CD105^–^CD133^–^ and CD14^+^DP refers to CD14^+^CD105^+^CD133^+^ (myeloid-derived pro-angiogenic cells; PAC).

Supplementary Figure 3 There is no clear phenotypic distinction between myeloid-derived PAC from patients with T2DM without retinopathy (NoDR), patients with T2DM with non-proliferative diabetic retinopathy (NPDR) and patients with T2DM with proliferative diabetic retinopathy (PDR). Principal component analysis (PCA) based on median fluorescent intensities (MFI) of all markers as measured on PAC from HC (n = 11), patients with NoDR (n = 9) patients with T2DM wih NPDR (n = 17), and patients with PDR (n = 7). The PCA plot shows the multivariate variation among the control (green), NoDR (blue), NPDR (purple) and PDR (red) groups. Small dots represent each donor, and the large dots show the average of each group. The first two principal axes explained 32.7% and 28.3% of the variance.

Supplementary Table 1 Interpretation of the cell surface markers detected on PAC.

## References

[1] Costa PZ Soares R. Neovascularization in diabetes and its complications. unraveling the angiogenic paradox. Life Sci (2013) 92(22):1037–45. doi: 10.1016/j.lfs.2013.04.001 23603139

[2] Jaipersad AS Lip GYH Silverman S Shantsila E. The role of monocytes in angiogenesis and atherosclerosis. J Am Coll Cardiol (2014) 63(1):1–11. doi: 10.1016/j.jacc.2013.09.019 24140662

[3] Hernandez GE Iruela-Arispe ML. The many flavors of monocyte/macrophage–endothelial cell interactions. Curr Opin Hematol (2020) 27(3):181–9. doi: 10.1097/MOH.0000000000000573 PMC7717488 32167947

[4] Choi Y-E Cha YR Lee K-m Kim HJ Yoon C-H. Proangiogenic cells enhanced persistent and physiologic neovascularization compared with macrophages. Exp Mol Med (2015) 47:e186. doi: 10.1038/emm.2015.60 26403262 PMC4650932

[5] Asosingh K Aldred MA Vasanji A Drazba J Sharp J Farver C. Circulating angiogenic precursors in idiopathic pulmonary arterial hypertension. Am J Pathol (2008) 172(3):615–27. doi: 10.2353/ajpath.2008.070705 PMC2258264 18258847

[6] Hildbrand P Cirulli V Prinsen RC Smith KA Torbett BE Salomon DR. The role of angiopoietins in the development of endothelial cells from cord blood CD34+ progenitors. Blood (2004) 104(7):2010–9. doi: 10.1182/blood-2003-12-4219 15213103

[7] Yoder MC. Endothelial progenitor cell: a blood cell by many other names may serve similar functions. J Mol Med (Berl) (2013) 91(3):285–95. doi: 10.1007/s00109-013-1002-8 PMC3704045 23371317

[8] Kalka C Masuda H Takahashi T Kalka-Moll WM Silver M Kearney M. Transplantation of ex vivo expanded endothelial progenitor cells for therapeutic neovascularization. Proc Natl Acad Sci U.S.A. (2000) 97(7):3422–7. doi: 10.1073/pnas.97.7.3422 PMC16255 10725398

[9] Özcan B Leenen PJM Delhanty PJD Baldéon-Rojas LY Neggers SJ van der Lely AJ. Unacylated ghrelin modulates circulating angiogenic cell number in insulin-resistant states. Diabetol Metab Syndr (2017) 9(1):43. doi: 10.1186/s13098-017-0239-8 28572856 PMC5452348

[10] Yoder MC Mead LE Prater D Krier TR Mroueh KN Li F. Redefining endothelial progenitor cells *via* clonal analysis and hematopoietic stem/progenitor cell principals. Blood (2007) 109(5):1801–9. doi: 10.1182/blood-2006-08-043471 PMC1801067 17053059

[11] Rose JA Erzurum S Asosingh K. Biology and flow cytometry of proangiogenic hematopoietic progenitors cells. Cytometry A (2015) 87(1):5–19. doi: 10.1002/cyto.a.22596 25418030 PMC4356492

[12] Pysna A Bem R Nemcova A Fejfarova V Jirkovska A Hazdrova J. Endothelial progenitor cells biology in diabetes mellitus and peripheral arterial disease and their therapeutic potential. Stem Cell Rev Rep (2019) 15(2):157–65. doi: 10.1007/s12015-018-9863-4 30413930

[13] Duong HT Erzurum SC Asosingh K. Pro-angiogenic hematopoietic progenitor cells and endothelial colony-forming cells in pathological angiogenesis of bronchial and pulmonary circulation. Angiogenesis (2011) 14(4):411–22. doi: 10.1007/s10456-011-9228-y PMC3725463 21796417

[14] Ghanchi F. Diabetic retinopathy guidelines working g. the royal college of ophthalmologists' clinical guidelines for diabetic retinopathy: A summary. Eye (Lond) (2013) 27(2):285–7. doi: 10.1038/eye.2012.287 PMC3574265 23306724

[15] Nilsson C Aboud S Karlén K Hejdeman B Urassa W Biberfeld G. Optimal blood mononuclear cell isolation procedures for gamma interferon enzyme-linked immunospot testing of healthy Swedish and Tanzanian subjects. Clin Vaccine Immunol (2008) 15(4):585–9. doi: 10.1128/CVI.00161-07 PMC2292660 18287577

[16] Shariatzadeh M Brandt MM Cheng C Ten Berge JC Rothova A Leenen PJM. Three-dimensional tubule formation assay as therapeutic screening model for ocular microvascular disorders. Eye (Lond) (2018) 32(8):1380–6. doi: 10.1038/s41433-018-0089-0 PMC6085384 29743587

[17] Forrester JV Kuffova L Delibegovic M. The role of inflammation in diabetic retinopathy. Front Immunol (2020) 11. doi: 10.3389/fimmu.2020.583687 PMC7677305 33240272

[18] Tepper OM Galiano RD Capla JM Kalka C Gagne PJ Jacobowitz GR. Human endothelial progenitor exhibit impaired proliferation, cells from type II diabetics adhesion, and incorporation into vascular structures. Circulation (2002) 106(22):2781–6. doi: 10.1161/01.CIR.0000039526.42991.93 12451003

[19] Terenzi DC Al-Omran M Quan A Teoh H Verma S Hess DA. Circulating pro-vascular progenitor cell depletion during type 2 diabetes: Translational insights into the prevention of ischemic complications in diabetes. JACC Basic Transl Sci (2018) 4(1):98–112. doi: 10.1016/j.jacbts.2018.10.005 30847424 PMC6390504

[20] Aitcheson SM Frentiu FD Hurn SE Edwards K Murray RZ. Skin wound healing: Normal macrophage function and macrophage dysfunction in diabetic wounds. Molecules (2021) 26:16. doi: 10.3390/molecules26164917 PMC8398285 34443506

[21] van Ark J Moser J Lexis CPH Bekkema F Pop I van der Horst ICC. Type 2 diabetes mellitus is associated with an imbalance in circulating endothelial and smooth muscle progenitor cell numbers. Diabetologia (2012) 55(9):2501–12. doi: 10.1007/s00125-012-2590-5 PMC3411291 22648662

[22] Tecilazich F Dinh T Pradhan-Nabzdyk L Leal E Tellechea A Kafanas A. Role of endothelial progenitor cells and inflammatory cytokines in healing of diabetic foot ulcers. PloS One (2013) 8(12):e83314–e. doi: 10.1371/journal.pone.0083314 PMC3865213 24358275

[23] Jin Y Sharma A Carey C Hopkins D Wang X Robertson DG. The expression of inflammatory genes is upregulated in peripheral blood of patients with type 1 diabetes. Diabetes Care (2013) 36(9):2794–802. doi: 10.2337/dc12-1986 PMC3747909 23637351

[24] Kim SJ Chae S Kim H Mun DG Back S Choi HY. A protein profile of visceral adipose tissues linked to early pathogenesis of type 2 diabetes mellitus. Mol Cell Proteomics (2014) 13(3):811–22. doi: 10.1074/mcp.M113.035501 PMC3945910 24403596

[25] Lim RR Vaidya T Gadde SG Yadav NK Sethu S Hainsworth DP. Correlation between systemic S100A8 and S100A9 levels and severity of diabetic retinopathy in patients with type 2 diabetes mellitus. Diabetes Metab Syndr (2019) 13(2):1581–9. doi: 10.1016/j.dsx.2019.03.014 31336525

[26] Averill MM Kerkhoff C Bornfeldt KE. S100A8 and S100A9 in cardiovascular biology and disease. Arterioscler Thromb Vasc Biol (2012) 32(2):223–9. doi: 10.1161/ATVBAHA.111.236927 PMC3262097 22095980

[27] Bouma G Coppens JM Lam-Tse WK Luini W Sintnicolaas K Levering WH. An increased MRP8/14 expression and adhesion, but a decreased migration towards proinflammatory chemokines of type 1 diabetes monocytes. Clin Exp Immunol (2005) 141(3):509–17. doi: 10.1111/j.1365-2249.2005.02865.x PMC1809454 16045741

[28] Burkhardt K Schwarz S Pan C Stelter F Kotliar K Von Eynatten M. Myeloid-related protein 8/14 complex describes microcirculatory alterations in patients with type 2 diabetes and nephropathy. Cardiovasc Diabetol (2009) 8:10. doi: 10.1186/1475-2840-8-10 19232095 PMC2654885

[29] Ahn GO Seita J Hong BJ Kim YE Bok S Lee CJ. Transcriptional activation of hypoxia-inducible factor-1 (HIF-1) in myeloid cells promotes angiogenesis through VEGF and S100A8. Proc Natl Acad Sci USA (2014) 111(7):2698–703. doi: 10.1073/pnas.1320243111 PMC3932909 24497508

[30] Li C Li S Jia C Yang L Song Z Wang Y. Low concentration of S100A8/9 promotes angiogenesis-related activity of vascular endothelial cells: bridges among inflammation, angiogenesis, and tumorigenesis? Mediators Inflammation (2012) 2012:248574. doi: 10.1155/2012/248574 PMC3363068 22685372

[31] Zhong X Xie F Chen L Liu Z Wang Q. S100A8 and S100A9 promote endothelial cell activation through the RAGEmediated mammalian target of rapamycin complex 2 pathway. Mol Med Rep (2020) 22(6):5293–303. doi: 10.3892/mmr.2020.11595 PMC7646991 33174028

[32] Landers-Ramos RQ Sapp RM VandeWater E Macko J Robinson S Wang Y. Investigating the extremes of the continuum of paracrine functions in CD34-/CD31+ CACs across diverse populations. Am J Physiol Heart Circ Physiol (2017) 312(1):H162–H72. doi: 10.1152/ajpheart.00342.2016 PMC5283912 27793853

[33] Lopes-Coelho F Silva F Gouveia-Fernandes S Martins C Lopes N Domingues G. Monocytes as endothelial progenitor cells (EPCs), another brick in the wall to disentangle tumor angiogenesis. Cells (2020) 9(1):107. doi: 10.3390/cells9010107 31906296 PMC7016533

[34] Rehman J Li J Orschell CM March KL. Peripheral blood "endothelial progenitor cells" are derived from monocyte/macrophages and secrete angiogenic growth factors. Circulation (2003) 107(8):1164–9. doi: 10.1161/01.CIR.0000058702.69484.A0 12615796

[35] Peplow PV. Growth factor- and cytokine-stimulated endothelial progenitor cells in post-ischemic cerebral neovascularization. Neural Regener Res (2014) 9(15):1425–9. doi: 10.4103/1673-5374.139457 PMC4192942 25317152

[36] He T Smith LA Harrington S Nath KA Caplice NM Katusic ZS. Transplantation of circulating endothelial progenitor cells restores endothelial function of denuded rabbit carotid arteries. Stroke (2004) 35:10. doi: 10.1161/01.STR.0000141893.33677.5d 15345801

[37] Medina RJ O’Neill CL O’Doherty TM Knott H Guduric-Fuchs J Gardiner TA. Myeloid angiogenic cells act as alternative M2 macrophages and modulate angiogenesis through interleukin-8. Mol Med (2011) 17(9):1045–55. doi: 10.2119/molmed.2011.00129 PMC3188859 21670847

[38] Isner JM Asahara T. Angiogenesis and vasculogenesis as therapeutic strategies for postnatal neovascularization. J Clin Invest (1999) 103(9):1231–6. doi: 10.1172/JCI6889 PMC408362 10225965

[39] Fadini GP Sartore S Albiero M Baesso I Murphy E Menegolo M. Number and function of endothelial progenitor cells as a marker of severity for diabetic vasculopathy. Arterioscler Thromb Vasc Biol (2006) 26(9):2140–6. doi: 10.1161/01.ATV.0000237750.44469.88 16857948

