# Supplementary figures and images for: Aberration in myeloid-derived pro-angiogenic cells in type-2 diabetes mellitus; implication for diabetic retinopathy?
Source: Front Ophthalmol (Lausanne). 2023 Mar 23;3:1119050. doi: 10.3389/fopht.2023.1119050 (PMC11182312; doi:10.3389/fopht.2023.1119050)

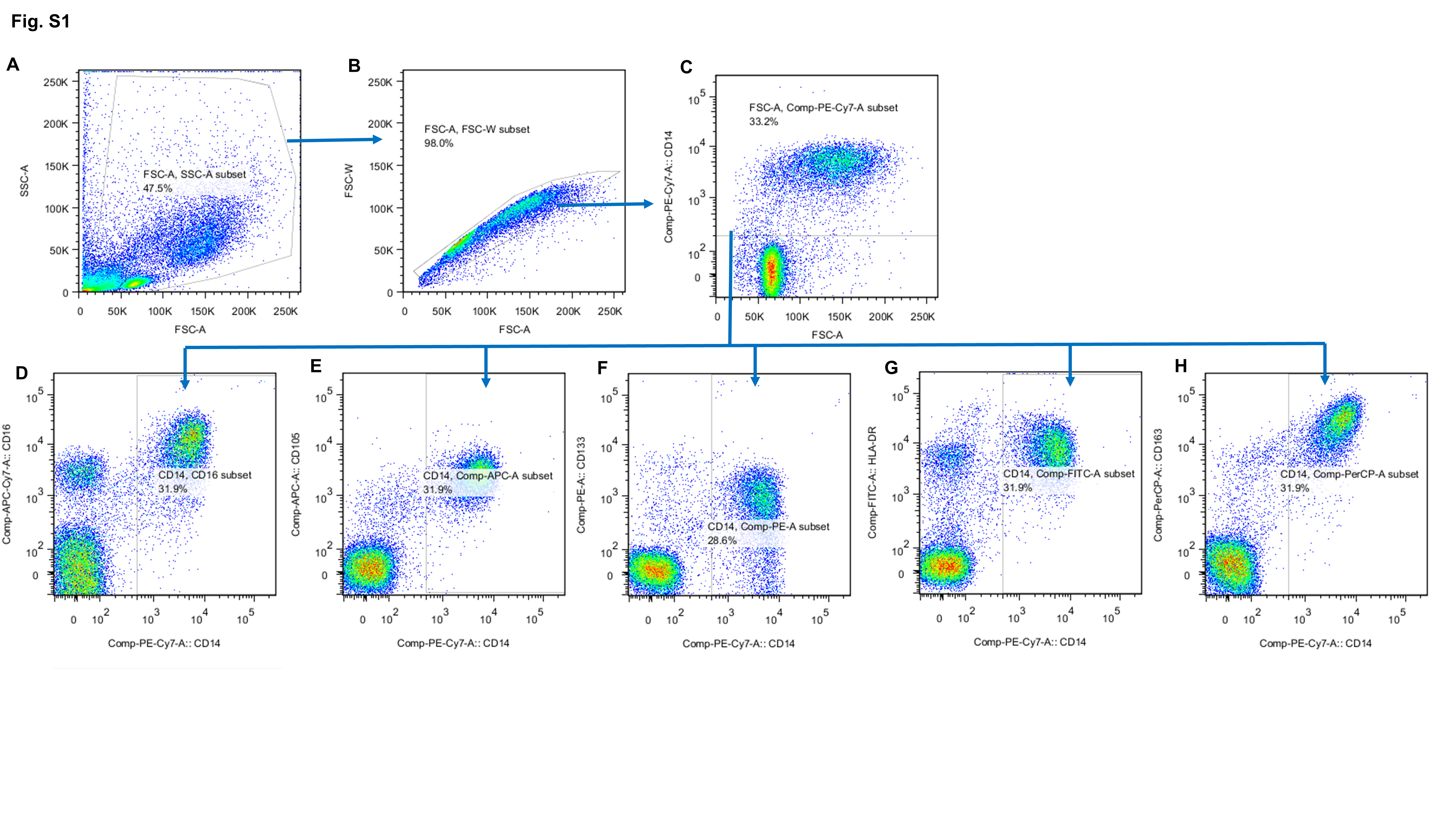

Supplement: Supplementary Figure 1 — Gating strategy for flow cytometric analysis of myeloid-derived pro-angiogenic cells (PAC). (A) Forward scatter (FSC) x side scatter (SSC) gating to obtain mononuclear cells based on size and granularity and exclude debris. (B) cell aggregate exclusion. (C) gate for CD14+ monocyte-derived cells. (D-H) The representative marker expressions are plotted against the CD14 expression. Identical gates were used for auto-fluorescence compensation. [file Image_1.tif]

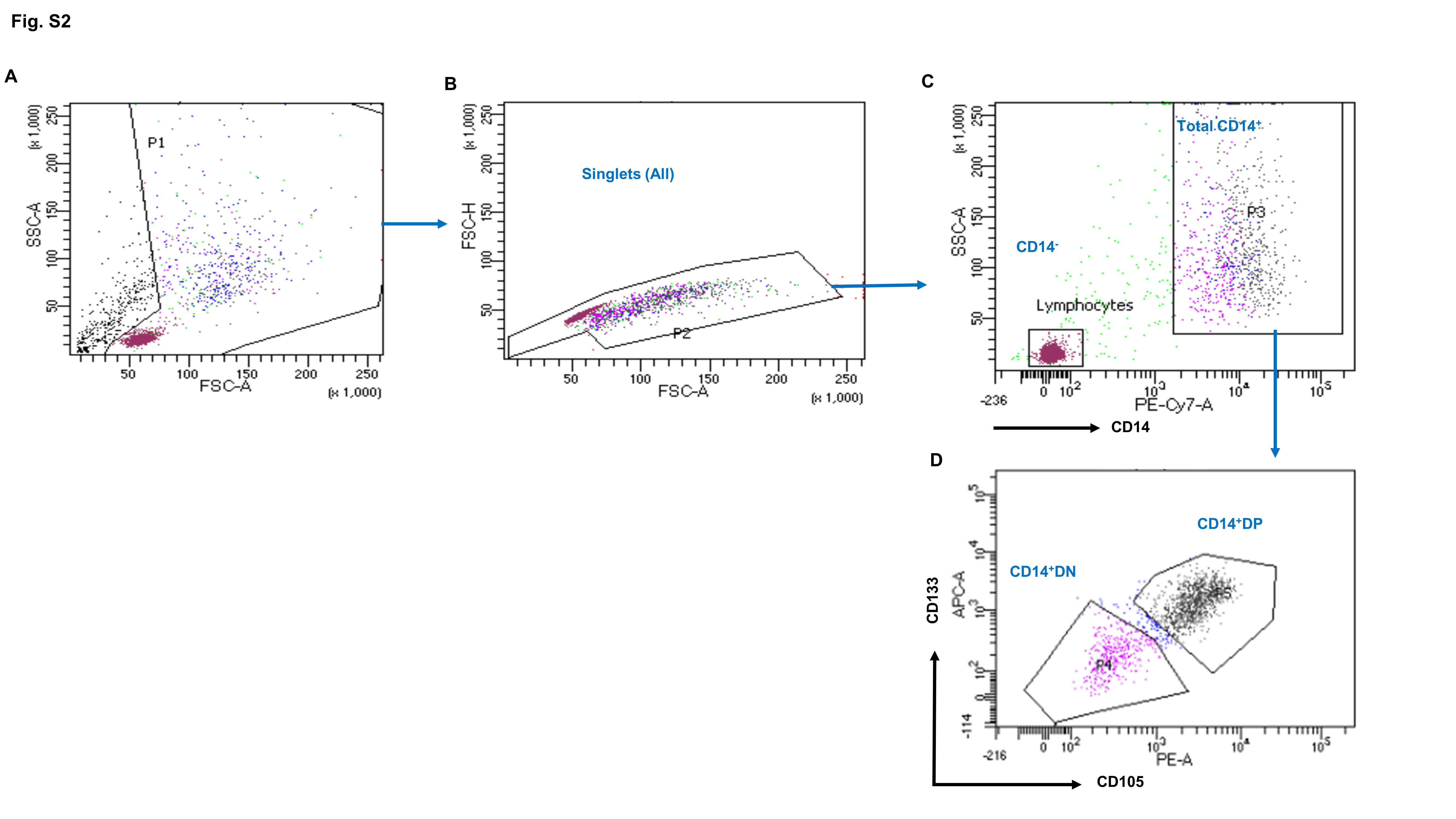

Supplement: Supplementary Figure 2 — Gating strategy for sorting cell subsets from PAC cultures. (A) Forward scatter (FSC) x side scatter (SSC) gating to obtain mononuclear cells based on size and granularity (P1). (B) Cell aggregate exclusion (P2). (C) Outline of gating strategy for myeloid and lymphoid cells. (D) gates for CD14+ cell subsets based on CD105 and CD133. CD14+DN refers to CD14+CD105–CD133– and CD14+DP refers to CD14+CD105+CD133+ (myeloid-derived pro-angiogenic cells; PAC). [file Image_2.tif]

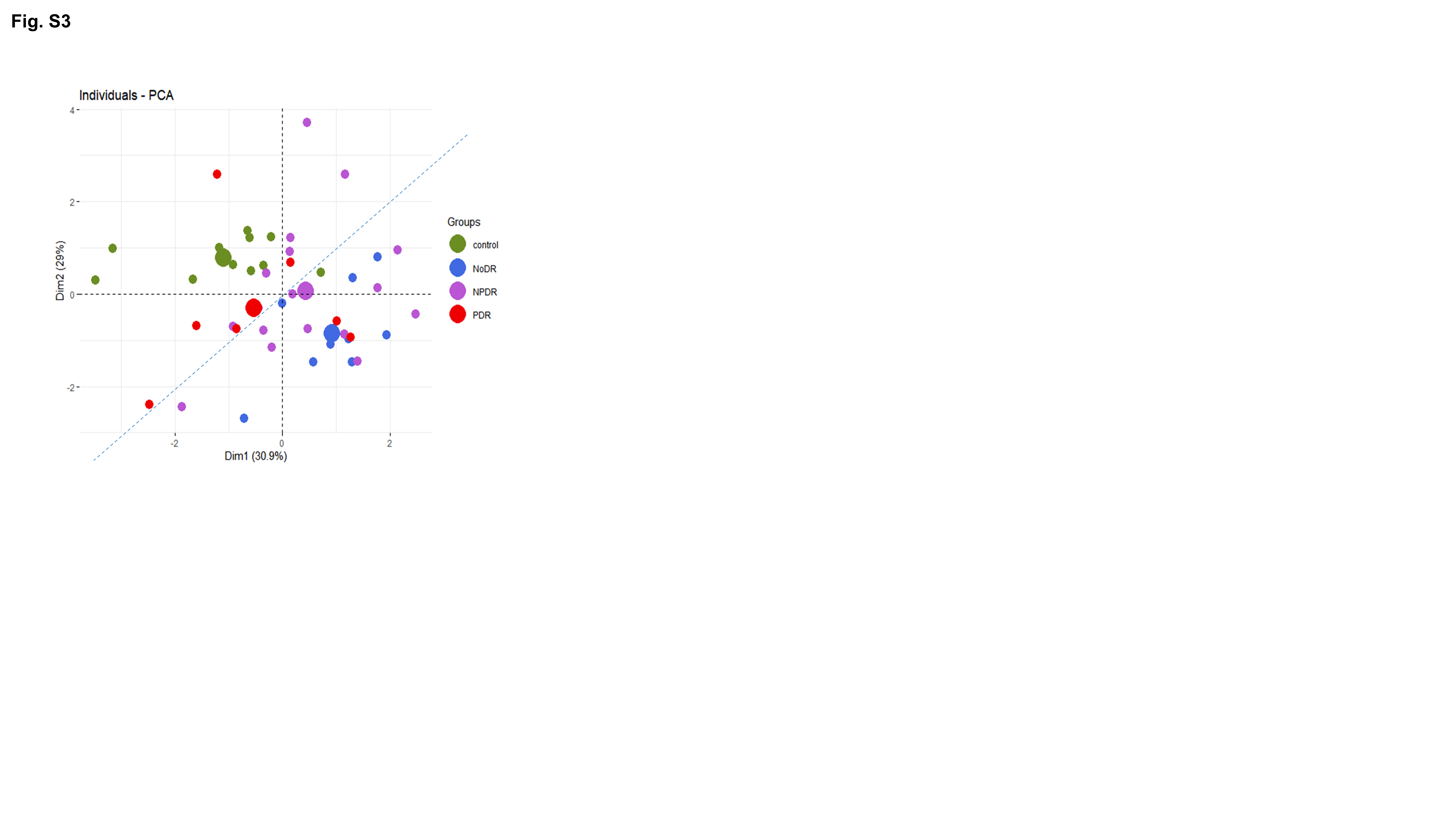

Supplement: Supplementary Figure 3 — There is no clear phenotypic distinction between myeloid-derived PAC from patients with T2DM without retinopathy (NoDR), patients with T2DM with non-proliferative diabetic retinopathy (NPDR) and patients with T2DM with proliferative diabetic retinopathy (PDR). Principal component analysis (PCA) based on median fluorescent intensities (MFI) of all markers as measured on PAC from HC (n = 11), patients with NoDR (n = 9) patients with T2DM wih NPDR (n = 17), and patients with PDR (n = 7). The PCA plot shows the multivariate variation among the control (green), NoDR (blue), NPDR (purple) and PDR (red) groups. Small dots represent each donor, and the large dots show the average of each group. The first two principal axes explained 32.7% and 28.3% of the variance. [file Image_3.tif]
