# Supplementary material for: Aberration in myeloid-derived pro-angiogenic cells in type-2 diabetes mellitus; implication for diabetic retinopathy?
Source: Front Ophthalmol (Lausanne). 2023 Mar 23;3:1119050. doi: 10.3389/fopht.2023.1119050 (PMC11182312; doi:10.3389/fopht.2023.1119050)
Supplement: Supplementary Table 1 — Interpretation of the cell surface markers detected on PAC. [file Table_1.pdf]

Table S1

Surface markers	Molecular identity	Indication
**CD14**	LPS co-receptor	Monocyte/macrophage (M1) marker
**CD16**	FcγRIII	Monocyte/macrophage marker (↑ expression by M2-like stimulation)
**CD105**	Endoglin	Endothelial cell marker
**CD133**	Prominin-1	Stem cell marker
**CD31**	PECAM-1	Endothelial cell marker
**HLA-DR**	MHC-II	Antigen presenting cells activation marker
**CD163**	scavenger receptor for the hemoglobin-haptoglobin complex	Alternatively activated macrophages marker
**CD206**	Mannose receptor	Alternatively activated macrophages marker
